# Fourier Power Spectrum Characteristics of Face Photographs: Attractiveness Perception Depends on Low-Level Image Properties

**DOI:** 10.1371/journal.pone.0122801

**Published:** 2015-04-02

**Authors:** Claudia Menzel, Gregor U. Hayn-Leichsenring, Oliver Langner, Holger Wiese, Christoph Redies

**Affiliations:** 1 Person Perception Research Unit, Friedrich-Schiller-University Jena, Jena, Germany; 2 Experimental Aesthetics Group, Institute of Anatomy I, Jena University Hospital, Friedrich-Schiller-University Jena, Jena, Germany; 3 Department of Neurology, University of Lübeck, Lübeck, Germany; 4 Department of Psychology, Durham University, Durham, United Kingdom; University of Melbourne, AUSTRALIA

## Abstract

We investigated whether low-level processed image properties that are shared by natural scenes and artworks – but not veridical face photographs – affect the perception of facial attractiveness and age. Specifically, we considered the slope of the radially averaged Fourier power spectrum in a log-log plot. This slope is a measure of the distribution of special frequency power in an image. Images of natural scenes and artworks possess – compared to face images – a relatively shallow slope (i.e., increased high spatial frequency power). Since aesthetic perception might be based on the efficient processing of images with natural scene statistics, we assumed that the perception of facial attractiveness might also be affected by these properties. We calculated Fourier slope and other beauty-associated measurements in face images and correlated them with ratings of attractiveness and age of the depicted persons (Study 1). We found that Fourier slope – in contrast to the other tested image properties – did not predict attractiveness ratings when we controlled for age. In Study 2A, we overlaid face images with random-phase patterns with different statistics. Patterns with a slope similar to those in natural scenes and artworks resulted in lower attractiveness and higher age ratings. In Studies 2B and 2C, we directly manipulated the Fourier slope of face images and found that images with shallower slopes were rated as more attractive. Additionally, attractiveness of unaltered faces was affected by the Fourier slope of a random-phase background (Study 3). Faces in front of backgrounds with statistics similar to natural scenes and faces were rated as more attractive. We conclude that facial attractiveness ratings are affected by specific image properties. An explanation might be the efficient coding hypothesis.

## Introduction

The perception and assessment of beauty influences humans every day (e.g., [1–3]). The scientific exploration of aesthetics traces back to the German psychologist Gustav Theodor Fechner [4], who is considered the founder of psychophysics and experimental aesthetics. Psychophysics describes laws that relate physical stimuli to the corresponding percept. Related to this, experimental aesthetics aims at discovering objective criteria, e.g., physical properties of stimuli that are commonly considered as beautiful or attractive. Among the great variety of visual stimuli that we regularly evaluate according to their beauty, the human face is the most relevant for social interactions.

Although the variability in attractiveness ratings suggests that beauty standards differ between observers to a certain degree [5, 6], several researchers have argued that evaluations on attractiveness are surprisingly consistent across observers, social groups and even cultures (e.g., [3, 7–9]). Here, we distinguish between two kinds of characteristics affecting the aesthetic perception of faces (and images): First, morphological properties of the face and, second, low-level properties of the image structure. Although the two types of property are related to each other, their distinction seems useful to classify the different research directions. Some of the well-known morphological properties that have been shown to correlate with facial attractiveness are symmetry, averageness, and sexual dimorphism (as reviewed by [8–10]). Besides these better-known beauty characteristics, low-level properties of face images also have an impact on the evaluation of facial attractiveness. For example, skin colour and texture affect the evaluation of facial attractiveness (e.g., [11–14]). For female faces in particular, an enhanced perceived attractiveness was found for increased facial contrast, which can be induced by using make-up [15].

Obviously, several natural changes to the face affect both morphological and low-level properties. Among these properties, age is important for social evaluation and related to attractiveness. With increasing age, morphological characteristics change (as reviewed by [16, 17]). Relevant for our study, attractiveness is influenced by age-associated changes of the skin, especially for female faces [11, 18–20]. Two studies that investigated age perception of skin patches showed that age estimations correlated with statistical image properties, such as homogeneity, colorimetric and light diffusion parameters, of the skin patches [21, 22].

A frequently studied low-level image property of human faces is their spatial frequency (SF) content. The usage of high spatial frequencies (HSFs) and low spatial frequencies (LSFs) depends on the respective face processing task [23]. Overall, the significance of specific frequency bands is still a matter of scientific debate. Although it has been hypothesized that LSFs are more important for holistic face recognition [24], more recent studies provide evidence that also HSFs contribute to holistic face processing to a similar degree [25]. Furthermore, Ruiz-Soler and Beltran [23] hypothesize that some people prefer to process HSFs, while others prefer LSFs. Nevertheless, medium SFs (8–16 cycles per face width) are considered to be crucial for face identification [23], while the HSF band plays an important role in the categorization of face gender and expression [26]. Interestingly, from a neurophysiological perspective, it has been suggested that LSFs and HSFs are processed in separate brain pathways that converge in the right fusiform gyrus [27].

Here, we focus on the relative distribution of spatial frequency power within a given image and its relation to facial attractiveness. The importance of the frequency composition for aesthetic perception has been demonstrated for different kinds of aesthetically pleasing images: Images of complex natural scenes and artworks both exhibit specific patterns in their frequency distribution [28, 29]. Additionally, images with frequency distributions that systematically deviate from those of natural scenes may induce visual discomfort [30–32].

Our method of choice for determining the spatial frequency distributions of images is the Fourier transformation. The relative distribution of frequency power is usually expressed by describing the relation between spatial frequency and power of the radially averaged (1d) Fourier spectrum on a log-log scale. For most natural images, this relation is linear, falling off in accordance to a power law. The slope of this linear function indicates the relative strength (i.e., power) of fine detail (HSFs) and coarse structures (LSFs) in an image. Increased power of HSFs results in shallower slopes whereas enhanced LSFs lead to steeper slopes. Most importantly, the frequency distribution of complex natural scenes is characterized by a slope of approximately -2 in power plots (corresponds to -1 in amplitude plots), implying that these images possess a scale-invariant spatial frequency spectrum with the same total energy (e.g., contrast) at every scale [33–35]. Interestingly, large subsets of artworks and other visually pleasing images share this Fourier power slope of about -2 with natural scenes [28, 29, 36–38]. Face photographs generally possess slopes steeper than those of natural scenes [39, 40]. Nevertheless, many artists portray human faces with a frequency composition more similar to those of natural scenes [40]. Therefore, we hypothesize that a Fourier slope of -2 is generally preferred by humans [41] and that face images with this slope (or with a slope shifted in the direction of -2) will be considered as more attractive by the observers.

Besides the Fourier transform, there are other measures for low-level image properties that can be associated with aesthetical experience. A measure closely related to scale-invariance is self-similarity [42]. High self-similarity implies that an image as a whole has an appearance similar to its parts. One method to calculate self-similarity is the Pyramid of Histograms of Orientation Gradients (PHOG) method ([43, 44]; for details, see Appendix in [36] and the Image analysis section in Study 1A).

Another low-level image measure that has been associated with aesthetic perception is image complexity which relates to the number and irregularity of elements that an image is composed of (for a review see [45]). Berlyne [46] postulated that a high aesthetic appeal is linked to an intermediate level of complexity. This notion is still considered valid today (for a review, see [45]), although the range of complexity values observed in artworks and other aesthetic images is rather wide [42].

Finally, we measured anisotropy which represents the degree, to which the strength of oriented gradients within an image differ between orientations (see [38, 42]). In veridical faces, horizontal contours are of importance for face identification, identity after-effects, and holistic face processing [47–49]. Moreover, artists tend to create artworks with a more isotropic Fourier spectrum than their corresponding real-world model [38]. Similarly, Redies et al. [42] described that overall gradient strength is more uniformly distributed across orientations in large subsets of coloured artworks of Western provenance compared to other categories of images. The perceptual significance of these findings remains unclear at present.

In view of the relation between the low-level properties of images and their aesthetic reception outlined above, we hypothesized that facial attractiveness, as an aesthetic dimension, may be related to the Fourier slope and other low-level measures of the face images. Typically, attractiveness is presumed to be primarily a dimension of the morphology of a person rather than a dimension influenced by image statistics. Previous research, however, indicates that low-level properties of face stimuli may nevertheless play an important role for beauty and aesthetics. In an ERP-study, Blickhan and colleagues modified the Fourier slope of face images and found that enhancing the power of LSFs (i.e., a steeper slope) led to impaired face learning, whereas an enhancement of HSF power (i.e., a shallower slope, similar to those of natural scenes) led to a facilitation of neuronal correlates of face learning [50]. This suggests that a modification of the image statistics can enhance face processing. Additionally, it has been shown that scale-invariant images with a Fourier slope of about -2 are processed efficiently by the visual system [51, 52]. Therefore, aesthetic experience might correlate with the efficient sensory coding of images that possess natural scenes statistics [41]. Interestingly, other research suggests that such images may be processed more fluently in the brain [53].

In conclusion, to gain more insight into the possible basis for low-level processing in face perception, we studied statistical properties of face images in relation to perceived facial attractiveness. Here, we report three main studies, in which we correlated image statistics with ratings of attractiveness, gathered ratings for images with directly manipulated image statistics, and looked at the influence of image backgrounds with particular image statistics, respectively. To this aim, we performed a Fourier transformation and a PHOG analysis in order to measure the properties of the Fourier power spectrum, as well as PHOG self-similarity, complexity and anisotropy of luminance gradients (Study 1). We hypothesized that facial attractiveness correlates (when controlled for a potential age effect) with these statistical image properties. Specifically, we hypothesized that veridical images of faces that are rated as more attractive possess a shallower Fourier slope (i.e., a slope closer to -2) and are more self-similar compared to less attractive faces. We expected an effect of face gender since, for example, facial contrast enhances female but decreases male attractiveness ratings [15]. Higher complexity due to inhomogeneous skin and/or beard stubble might interact with gender as well. In Studies 2 and 3, we focussed on properties derived from the Fourier transform. By manipulating the spectral slope, we modified the spatial frequency spectrum in face images in order to investigate the degree of change in perceived facial attractiveness and age (Study 2). Here, we hypothesized that modified face images with a slope similar to natural scenes and artworks (i.e., about -2) would be preferred. Compared to the band-pass filtered images used in previous studies (e.g., [25, 54]), slope-manipulated images have the advantage of looking more natural [50]. In our last study (Study 3), we did not alter the face images but superimposed them on backgrounds with different statistics. The aim of this study was to investigate whether background features influence the attractiveness ratings as well. We expected that facial attractiveness would be rated highest on backgrounds with statistics similar to natural scenes because these images might be processed more efficiently and/or fluently.

We found correlations between facial attractiveness perception and three out of four image statistics investigated. Manipulations of the face images or their backgrounds, respectively, had an effect on perceived facial attractiveness (and age). Our study, thus, identifies image properties that relate to distinct facial characteristics (attractiveness and age) and may be detected with high speed at low levels of face processing.

## Study 1

In the first study, we ran linear models to investigate possible correlations between different low-level image properties and attractiveness. We also controlled for the effect of age of the depicted person since age-related changes correlate with attractiveness perception [11, 55–58]. To this aim, we calculated several statistical image properties of face photographs from different available face databases. In Study 1A, we asked participants to spontaneously rate images from the FACES database according to the attractiveness of the face [59]. In Study 1B, we investigated existing behavioural data for a mixture of other image sets that had already been rated by participants in a previous experiment for their attractiveness and perceived age.

### Study 1A

#### Materials & Methods

*Ethical note*. All studies presented here were conducted in accordance with the ethical guidelines of the Declaration of Helsinki and were approved by the ethics committee of Jena University Hospital. The participants of all studies gave their written consent prior to the experiment. All participants reported normal or corrected-to-normal vision.

**Fig 1:**
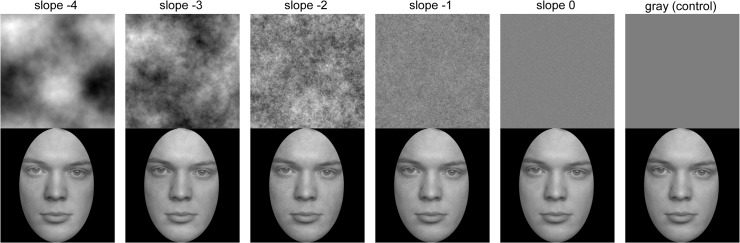
Stimuli used in Study 2A. Top row: Random phase patterns with different slopes (as indicated) of the radially averaged Fourier power spectrum, and a mid-grey control image. Bottom row: Stimuli used in Study 2A. The composite stimuli consists of the original face image (FACES database [59]) with the respective image of the top row superimposed at an opacity of 15% and a black oval window. Note that differences between the conditions are subtle and might be invisible due to the small size and low resolution of the images here. Images of higher resolution are provided as supplemental material (S1 Fig).

*Stimuli*. Stimuli were taken from the FACES database [59] and consisted of grey-scale digital photographs of 56 male and 54 female Caucasians who were between 19 and 55 years old (M = 36.23 years, SD = 13.09). All persons had been photographed in frontal view and had neutral facial expression with no specific features, such as glasses, extensive facial hair, ostentatious make-up, or jewellery. The images were resized to 1024 x 1024 pixels and grey-scaled. Then, faces were fitted behind a black oval window to hide hair, background and outer facial contours (similar to those in Fig 1, bottom row).

*Image analysis*. First, we analyzed self-similarity, complexity and anisotropy in 110 face images with a method that was originally derived from the PHOG descriptor [43], as described previously [36, 42]. A detailed account of this method can be found in the appendix of Braun et al. [36]. In brief, the size of all images was reduced to 100,000 pixels by bicubic interpolation and isotropic scaling. In the PHOG calculation, histograms of oriented luminance gradients (HOG features) [44] were compared at different levels of an image pyramid [60]. After calculating the HOG features for the entire image (level 0), the image was divided into four equally sized rectangles to yield level 1. Each section at level 1 was then again divided into four equally sized rectangles to obtain the level 2 of the pyramid (16 sections). At the third level, there are 64 sections. HOG features were calculated for each section at a given level. Histograms were obtained for 16 equal bins covering 360 degrees (full circle) [42]. In the present study, self-similarity was calculated as the mean of the self-similarity values for levels 1 to 3 of the pyramid compared to level 0. The scale ranges from 0 to 1 with 1 being a highly self-similar image. Complexity was calculated as the sum of the strengths of all gradients across all orientations at level 0. The variance of the luminance gradient strengths across the 16 orientation bins at level 3 of the pyramid was taken as a measure for anisotropy and indicates how much the strength of the oriented gradients differs across orientations. An almost uniform distribution of all orientations results in a value close to zero. All calculations were performed using MATLAB (MathWorks). For an analysis of correlations between the different PHOG measures and the Fourier slope value, see Braun et al. [36].

**Fig 2:**
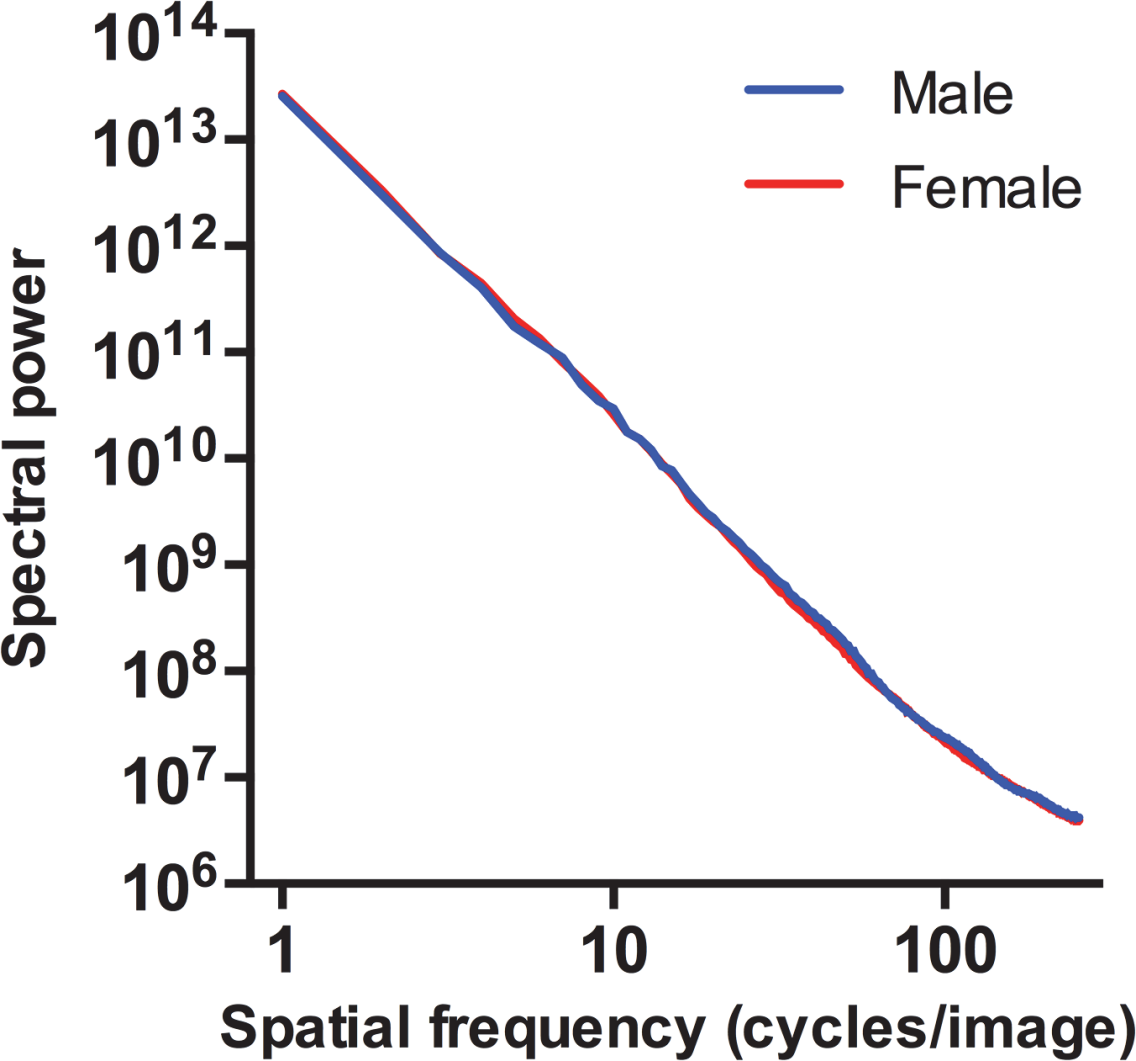
Log-log plot of radially averaged spectral power *versus* spatial frequency. Average curves are given for male and female face images from the dataset used in Study 1A (FACES database [59]).

Second, we determined the slope of the curve of the radially averaged spatial frequencies and their power in log-log plots, as done previously in research on natural scenes [33, 61] and artworks [28, 29, 40]. In brief, images were resized to 512 x 512 pixels by bicubic interpolation. Then, the discrete Fourier transform (2d Fast Fourier Transform) was computed to obtain the power spectrum. In contrast to several previous studies on spatial frequencies of face images (e.g., [54], see [39] for a discussion), we did not calculate cycles per face width but cycles per image in order to be able to perform standardized analyses and image modifications and to compare our results with previous studies [38, 40, 50]. Radially averaged power was plotted in the log-log plane as a function of spatial frequency (see Fig 2). To measure the slope of the resulting log-log frequency spectrum, data points were maximally binned at regular frequency intervals (i.e., 33 bins) in the log-log plane and a least-squares fit of a line was performed to the binned data points in the range of 10–255 cycles/image. This range was chosen for comparison with previous studies using a similar range to reduce effects of artefacts [38, 40]. The slope of the fitted line was then determined. All calculations were carried out using Python.

*Participants*. Forty participants (14 male, mean age = 21.1 years, range = 18–26 years) took part in the rating experiment. All participants were students or graduates of medical or life sciences in Germany.

*Procedure*. We instructed participants to rate each of the presented faces according to its attractiveness. Participants were told that attractiveness was defined as the physical allurement of a face and that the rating should reflect how pleasant they perceived it. In total, all 110 faces were presented in random order and rated once. In each trial, first a question mark was presented for 800ms, followed by the test image for 600ms, and a blank screen for 1600ms. Participants were asked to evaluate the attractiveness of the presented face on a rating scale by pressing one of four labelled keys on the computer keyboard. The scale reached from 1 (very attractive) to 4 (very unattractive), based on the German grading system. However, in order to create results comparable with the follow-up experiments (Studies 1B, 2A, 2B and 3A), we inverted the results leading to an artificial scale from 1 (very unattractive) to 4 (very attractive). We used solely this inverted scale within the manuscript and for our statistical analysis.

In all Studies with the exception of Study 1B, images were presented on a calibrated black screen (EIZO ColorEdge CG241W) at a constant viewing distance of 100cm, ensured by the use of a chin rest. The monitors where calibrated with a colorimeter (X-Rite EODIS3 i1Display Pro) using the same calibration profile in order to create similar conditions for all observers. Stimuli were presented at a size of 10 x 14.1cm, covering 5.7° x 8.4° degrees of visual angle. The experiments took place in a room with closed shutters, holding illumination at low background levels and constant over participants.

#### Results

Overall, the mean of rated attractiveness was 2.21 (SD = .57), with females rated more attractive than male faces (females: M = 2.36, SD = .58; males: M = 2.06, SD = .53; two-samples t-test: T(108) = -3.019, p < .01). Mean Fourier slope was -2.801 (SD = .10) with no difference between face gender (two-samples t-test: T(108) = .159, n.s.; Fig 2). Mean PHOG self-similarity was. 467 (SD = .03) with a tendency of higher values for male faces (two-samples t-test: T(108) = 1.791, p = .076; males: M = .473, SD = .03, females: M = .462, SD = .03). Mean complexity was 3.766 (SD = .36) with no differences between face gender (two-samples t-test: T(108) = -1.068, n.s.). Mean anisotropy was. 00135 (SD = .000080) with no differences between genders (two-samples t-test: T(108) = -1.078, n.s.).

Pearson correlations of attractiveness ratings and chronological age with all four tested image properties revealed significant relations (Table 1). Interestingly, chronological age of the depicted persons and rated attractiveness correlated negatively (Pearson r = -.769, p < .001). Since age also correlated with each of the image properties (Table 1), we ran second-level regression models to control for age as a confounding factor. Therefore, we centred data and calculated the residuals of the regression of each image property with chronological age of the faces. For each participant, we performed the following steps. First, the residuals of the regression of age and attractiveness rating were calculated. Then, we fitted a linear model to predict the residuals of the age-attractiveness regression from the residuals of the age-image property regressions. Finally, we conducted a one-sample t-test for each image property analysis using the regression coefficients of all participants to test whether they significantly differed from zero. This analysis allowed us to control for the confounding effect of age on attractiveness ratings and revealed the pure effect of the image property on rated attractiveness. A significant result from the t-test in this analysis revealed that the regression coefficients were unequal to zero and, thus, the tested image parameter predicted rated attractiveness.

**Table 1:** Pearson correlation coefficients of Study 1A (N_faces_ = 110; N_raters_ = 40).

	**rated attractiveness**	**chronological age**	**Fourier slope**	**PHOG self-similarity**	**PHOG complexity**
chronological age	-.769***				
Fourier slope	-.484***	.605***			
PHOG self-similarity	-.629***	.685***	.607***		
PHOG complexity	-.437***	.521***	.319***	.663***	
PHOG anisotropy	.398***	-.441***	-.500***	-.396***	-.239*

*** p < .001;

* p < .05

Coefficients for Fourier slope were not significantly different from zero (one-sample t-test: T(39) = -.904, n.s., mean β = -.105, range: -1.549 to 2.131). Fourier slope was lower for male compared to female faces (one-sample t-test: T(39) = -9.246, p < .0001, mean β = -.165, range:-.377 to. 059). There was no interaction of gender and Fourier slope (one-sample t-test: T(39) = -.128, n.s., mean β = -.016, range: -1.488 to 1.720).

Analysis of coefficients of PHOG self-similarity revealed a significant interaction with gender (one-sample t-test: T(39) = -2.254, p < .05, mean β = -1.086, range: -6.710 to 5.763). Self-similarity correlated negatively with attractiveness ratings of male—but not of female—faces (male faces: one-sample t-test: T(39) = -5.130, p < .0001, mean β = -3.064, range: -10.405 to 4.187; female faces: one-sample t-test: T(39) = -1.197, n.s., mean β = -.892, range: -11.885 to 8.360).

PHOG complexity interacted with gender (one-sample t-test: T(39) = 3.515, p < .01, mean β = .094, range:-.294 to. 457). Complexity correlated negatively with attractiveness ratings of female—but not male—faces (male faces: one-sample t-test: T(39) = -.834, n.s., mean β = -.032, range:-.552 to. 538; female faces: one-sample t-test: T(39) = -4.699, p < .0001, mean β = -.220, range: -1.058 to. 373).

Analysis of coefficients of PHOG anisotropy revealed a significant interaction with gender (one-sample t-test: T(39) = -5.088, p < .0001, mean β = -613.900, range: -2162.630 to 1110.339). Anisotropy correlated negatively with attractiveness ratings of male and positively with the ratings of female faces (male faces: one-sample t-test: T(39) = -2.532, p < .05, mean β = -451.400, range: -2762.645 to 2209.417; female faces: one-sample t-test: T(39) = 4.777, p < .0001, mean β = 776.400, range: 1723.861 to 2665.073).

A comparison of the statistical image properties revealed significant correlations between Fourier slope and PHOG self-similarity (Pearson r = .607, p < .001), PHOG complexity (Pearson r = .319, p < .001), and PHOG anisotropy (Pearson r = -.500, p < .001). Moreover, PHOG values correlated with each other as well (see Table 1 for details).

### Study 1B

#### Materials & Methods

*Stimuli*. In addition to the images of Study 1A, we analyzed another subset of coloured face images that had already been used and rated in a previous study [62]. This set consisted of 143 images of the CAL/PAL Face Database [63], 154 images of the FACES database [59] that were manipulated differently to the ones in Study 1A, 152 images of the FERET Database [64], 19 images of the Glasgow Unfamiliar Face Database [65] and 11 images of the A-Face Database [66]. All images were in colour and presented the face contour including hair in front of a black background. Noteworthy, some of the men wore beards. Lighting conditions differed between the different source databases included in this set of images. Altogether, this image set contained 479 faces (239 male and 240 female Caucasians between 18 and 80 years old; M = 43.01 years, SD = 18.04).

*Participants*. The studied population consisted of 24 undergraduate students (11 male; mean age = 22.3 years, range = 19–30 years). All participants reported normal or corrected to normal vision, and either received course credit or monetary reimbursement.

*Procedure*. The procedure of the original study gathering the ratings was as follows: Participants were seated in a dimly lit room with their heads in a chin rest that was positioned 87cm away from a computer screen. Face images were presented at an approximate viewing angle of 11.2° x 8.5°. Participants were asked to rate the age (from 00–99), attractiveness (from 1 = very unattractive to 6 = very attractive), and the distinctiveness (from 1 = not distinctive to 6 = very distinctive) of 532 face images. We only used the attractiveness and age ratings for our analysis. Images remained on the screen until a response was recorded, and participants were instructed to respond as spontaneously as possible.

*Image analysis*. We calculated self-similarity, complexity and anisotropy using the PHOG method described for Study 1A. We did not analyse the Fourier spectrum in this study because it is not possible so far to calculate these from exactly those images presented to the participants (i.e., colour versions of the face photographs that cannot be analyzed by standard Fourier transformation).

#### Results

Mean rating of attractiveness in Study 1B was 2.42 (SD = .57). There was no difference of attractiveness ratings between images depicting female or male faces (two-samples t-test: T (477) = -1.121, n.s.). Mean PHOG self-similarity was. 583 (SD = .04) with no difference between male and female faces (two-samples t-test: T(477) = -.835, n.s.). Mean complexity was 4.864 (SD = .84) with no difference between male and female faces (two-samples t-test: T(477) = -.422, n.s.). Mean anisotropy was. 000964 (SD = .000104) with no difference between face gender (two-samples t-test: T(477) = .0715, n.s.).

Pearson correlations of attractiveness ratings with all tested image properties revealed significant relations (Table 2). Chronological and rated age also correlated with two of the three tested image parameters (PHOG self-similarity and anisotropy; Table 2). Again, we ran second-level regression models to control for chronological age (see above, Study 1A). Additionally, by using the same procedure as for chronological age, we also controlled for the age ratings of each participant (Table 3).

**Table 2:** Pearson correlation coefficients of Study 1B (N_faces_ = 479; N_raters_ = 24).

	**rated attractiveness**	**chronological age**	**rated age**	**PHOG self-similarity**	**PHOG complexity**
chronological age	-.405***				
rated age	-.476***	.953***			
PHOG self-similarity	-.312***	.290***	.357***		
PHOG complexity	-.115*	n.s.	n.s.	.356***	
PHOG anisotropy	.212***	-.122**	-.167***	-.647***	-.485***

*** p < .001;

** p < .01;

* p < .05; n.s. = not significant

**Table 3:** Results of second-level regression models of Study 1B using rated age to control for age effect (N_faces_ = 479; N_raters_ = 24).

		PHOG self-similarity	PHOG complexity	PHOG anisotropy
image property	T	-7.261	-3.985	6.122
	mean β	-2.271***	-.071***	828.200***
	β range	-6.130 to. 108	-.252 to. 135	-590.117 to 2466.935
gender	T	.813	.884	.925
	mean β	.023 (n.s.)	.025 (n.s.)	.026 (n.s.)
	β range	-.333 to. 352	-.331 to. 355	-.330 to. 357
image property*gender	T	-1.041	-.106	-2.598
	mean β	-.174 (n.s.)	-.001 (n.s.)	-176.600* ^1^
	β range	-2.277 to 1.449	-.108 to. 089	-924.452 to 700.255

*** p < .001;

** p < .01;

* p < .05; n.s. = not significant;

^1^ mean β for male faces = 651.6***, mean β for female faces = 1005***

When controlling for chronological age of depicted persons, we obtained the following results. Coefficients for PHOG self-similarity were significantly below zero (one-sample t-test: T(23) = -8.110, p < .0001, mean β = -2.675, range: -6.823 to. 074). There was neither a difference between genders (one-sample t-test: T(23) = .985, n.s., mean β = .028, range:-.321 to. 355) nor an interaction of gender and self-similarity (one-sample t-test: T(23) = -.804, n.s., mean β = -.166, range: -2.517 to 1.665).

Coefficients for PHOG complexity were significantly below zero (one-sample t-test: T(23) = -4.718, p < .0001, mean β = -.090, range:-.259 to. 133). There was neither a difference between genders (one-sample t-test: T(23) = 1.116, n.s., mean β = .031, range:-.317 to. 358) nor an interaction of gender and complexity (one-sample t-test: T(23) = -.158, n.s., mean β = -.002, range:-.114 to. 093).

Analysis of coefficients of PHOG anisotropy revealed a significant interaction with gender (one-sample t-test: T(23) = -2.524, p < .05, mean β = -197.600, range: -1194.134 to 637.665). PHOG anisotropy correlated positively with attractiveness ratings of both genders but more strongly for female faces (male faces: one-sample t-test: T(23) = 4.885, p < .0001, mean β = 750.400, range: -625.207 to 2439.947; female faces: one-sample t-test: T(23) = 6.634, p < .0001, mean β = 1146.0, range: -802.211 to 2792.098).

We found similar results when the age rating of each participant was used (instead of the chronological age; Table 3).

### Discussion

In Study 1, rated attractiveness correlated negatively with Fourier slope (Study 1A only), PHOG self-similarity (Studies 1A and 1B) and complexity (Studies 1A and 1B), and positively with PHOG anisotropy (Studies 1A and 1B). Chronological (and in Study 1B also rated) age also correlated with the image properties—but in a direction opposite to the attractiveness ratings (Table 1 and 2). It has been shown previously that attractiveness ratings correlate negatively with age at least for female faces (see Results; [55–57], but see [67]). When controlled for the confounding variable age, our analyses revealed significant relations between rated attractiveness and the three PHOG measures. Fourier slope, however, did not predict attractiveness ratings when controlled for the age of the face. Since chronological age and attractiveness ratings correlated highly, it is likely that age conceals a potential effect of Fourier slope on attractiveness ratings. When controlled for age, Fourier slope differed between genders, with female face images possessing shallower slopes. Possibly, the hair visible in some female face images might increase the power of HSFs and therefore lead to a shallower slope. Generally, the investigated face images show a linear fall-off in the log-log plots of the Fourier power spectrum (Fig 2). This power-law behaviour indicates equal proportions of power at every scale. The linear fall-off also allows us to manipulate this slope in Study 2B and 2C (see below).

For PHOG self-similarity, we found that lower self-similarity correlated with higher attractiveness ratings, when age was controlled for. This relation was not found in Study 1A for female faces. Higher self-similarity might be elicited by an increase in skin blemishes that lead to a more similar distribution of gradients within the image. Thus, homogeneous skin might lead to lower self-similarity, and a homogeneous skin elicits higher attractiveness ratings (e.g., [11, 12, 58, 68]). The negative correlation of self-similarity with ratings of attractiveness was not expected. Since visual pleasing images share the property of high self-similarity [36, 42] we expected higher self-similarity in images of attractive faces.

The more complex the images were, the less attractive the face was rated (when controlled for age). This effect was found only for female faces but not for male faces in Study 1A. As mentioned above, inhomogeneous skin might lead to more gradients in the images and, consequently, to higher image complexity. Supposedly, there is not such an effect for men because the slightest indication of a beard or a light stubble (men in Study 1A did not wear extensive beards) would introduce more gradients, and a light stubble might lead to higher attractiveness ratings ([69] but also see [70]).

Female faces were rated as more attractive when PHOG anisotropy was high (when controlled for age). For male faces this relation was weaker (Study 1B) or inverse (Study 1A). Higher anisotropy means that gradients were less balanced across orientations in images. High anisotropy could potentially be elicited by a homogenous skin structure, in which the only strong gradients within the face image would the eyebrows and contours of face features; their gradients would not be balanced across orientations. Skin blemishes, however, introduce gradients (i.e., higher complexity, see above) that are more balanced across orientations. Perhaps for this reason, there was no relation between low anisotropy and high attractiveness ratings, as found for artworks [42].

Overall, we found correlations between three out of four tested statistical image measures and attractiveness ratings, as well as chronological age. However, the measured Fourier slope did not predict attractiveness ratings when the confounding age effect was controlled for. To further investigate the predicted relation between Fourier power spectrum characteristics and perceived attractiveness, we experimentally manipulated the Fourier slope in the following studies. We therefore manipulated face images and their background to investigate the influence of a modified Fourier slope on attractiveness (Studies 2 and 3) and age ratings (Study 2A). Besides Fourier slope, results from Study 1 regarding PHOG-derived measures are promising. However, we proceeded with an experimental manipulation of Fourier slope only because it is not possible to manipulate PHOG parameters in a reasonable manner so far. Note, however, that there is a high correlation between Fourier slope and PHOG self-similarity (Study 1A; [36]).

## Study 2

In the three experiments of Study 2, we manipulated the Fourier power spectrum characteristics of face photographs and investigated the effect on attractiveness perception.

In Study 2A, we investigated whether attractiveness and age perception is affected by superimposing specific statistical properties onto face images by overlaying them with random phase patterns. Based on the finding that visual artworks, including art portraits, and natural scenes share a Fourier slope of about -2 [28, 37, 40], we expected that faces were perceived as more attractive and younger when overlaid with patterns with a slope of -2 or images with an overall shallower slope (overlaid with patterns with a slope of -1 or 0). Two groups of participants rated attractiveness and age, respectively, of faces in modified images in independent rating sessions.

In Study 2B and 2C, we directly modified the Fourier power spectrum of face images (similar to [50]) to assess the effect on subjective attractiveness ratings. By directly manipulating the slope of the radially averaged log-log Fourier power spectrum, we changed the relation of HSF and LSF power only. This manipulation had no effect on overall spectral power and the phase of the spatial frequencies. In contrast to band-pass filtered images, changes were subtle and faces remained realistic. In Study 2B, subjects were asked to select the more attractive of two manipulated faces in a pairwise comparison. In Study 2C, participants manipulated the slope of the Fourier power spectrum directly and interactively until they reached a face image that was most attractive to them. We expected participants to prefer either the original face images or images with statistics more similar to natural scenes (shallower slopes) because they might be processed more efficiently and/or fluently [50, 51].

### Study 2A

#### Materials & Methods

*Participants*. Twenty participants (eight males, mean age = 22.9 years, range = 18–30 years), mainly students of medicine or life sciences, rated the attractiveness of faces. A different group of twenty-one medical or life science students (eight males, mean age = 22.6 years, range = 18–34 years) estimated the age of the same faces.

*Stimuli*. We used 120 grey-scaled face images from the FACES database (Study 1A, Fig 1). Images of 60 women and 60 men, were used (20 young, 20 middle-aged and 20 old individuals for each gender; Table 4).

**Table 4:** Chronological age of depicted individuals used in Study 2A.

	Total (N = 120)	Young (N = 40)	Middle-aged (N = 40)	Old (N = 40)
Total (N = 120)	19–80 (M = 48.53)	19–30 (M = 23.9)	43–55 (M = 49.03)	69–80 (M = 72.65)
Females (N = 60)	19–80 (M = 48.58)	19–30 (M = 23.55)	45–55 (M = 49)	69–80 (M = 73.2)
Males (N = 60)	20–77 (M = 48.47)	20–30 (M = 24.25)	43–55 (M = 49.05)	69–77 (M = 72.1)

Age data are presented in years.

As masks for the face images, we created random phase patterns with different Fourier statistics in MATLAB. Each mask image was generated by combining a power spectrum that was created with a desired slope and a random phase-spectrum, and by using an inverse Fourier transform to obtain to the corresponding real image. These images were additionally scaled to the intensity values of 0 to 255, leaving the desired slope of the power spectrum unchanged. For each of the slopes of -4, -3, -2, -1 and 0 (Fig 1), 120 random phase patterns were generated. In a control condition, a mask with a homogeneous medium grey value was superimposed onto each face image with 15% opacity. Each face image was combined with one randomly selected mask per category (i.e., 720 trials; mid-grey, five slope values). The faces were fitted behind a black oval window (Fig 1). The stimuli had a resolution of 1200 x 1200 pixels. After adding the mask and a black oval window, we presented each image at a visual angle of 5.7° x 8.4°.

A representative number of stimuli (each face with the grey control mask and with ten different random phase patterns per slope category, resulting in a total of 6120 images) was analyzed using Fourier transformation (see Study 1A). The mask had a relatively small but significant effect on the Fourier slope of the final image (Greenhouse-Geisser F(1.03, 122.1) = 5663.8, p < .0001, all post-hoc pairwise comparisons p < .0001; Table 5). Please note that here and for all further analyses, Greenhouse-Geisser correction was applied to all ANOVAs in which the assumption of sphericity was violated.

**Table 5:** Results of Fourier transformation of stimuli used in Studies 2A, 2B, 2C and 3A.

**Experiment**	**Condition**	**Measured Fourier slope**	**SD**
Study 2A	mask with a slope of -4	-2.915	.068
	mask with a slope of -3	-2.913	.068
	mask with a slope of -2	-2.872	.063
	mask with a slope of -1	-2.814	.057
	mask with a slope of 0	-2.821	.057
	mid-grey mask	-2.922	.066
Study 2B	steep slope	-3.038	.056
	original	-2.847	.062
	shallow slope	-2.585	.032
Study 2C	step 1	-3.882	.047
	step 25	-3.261	.038
	step 50	-2.573	.035
	step 75	-1.875	.043
	step 100	-1.209	.060
	original	-2.755	.116
	adjusted slope	-2.643	.224
Study 3A	background with a slope of -4	-2.932	.065
	background with a slope of -3	-2.871	.037
	background with a slope of -2	-1.971	.029
	background with a slope of -1	-1.299	.065
	background with a slope of 0	-1.254	.080

*Experimental design*. Participants were asked to rate the attractiveness or age, respectively, of each presented face on a mouse-based scale. The scale for the attractiveness rating seemed continuous (100 sub-steps) and endpoints were labelled with the German equivalent to ‘not attractive’ and ‘attractive’. For the age estimation, the rating scale ranged from 0 to 100 years. The continuous-looking scale was sub-divided into 10-year-steps. Before each rating, the cursor of the mouse was set to the midpoint of the scale.

Each participant ran 720 trials (for conditions, see above). First, a fixation cross was presented for a random duration between 300 and 800ms, followed by the stimulus on a black screen (800ms), and a blank screen with the rating scale. Participants had the time to respond to the rating scale for as long as they needed. After 60 trials, the participants were allowed to take a break for as long as they liked, before evaluating the next images.

#### Results

**Fig 3:**
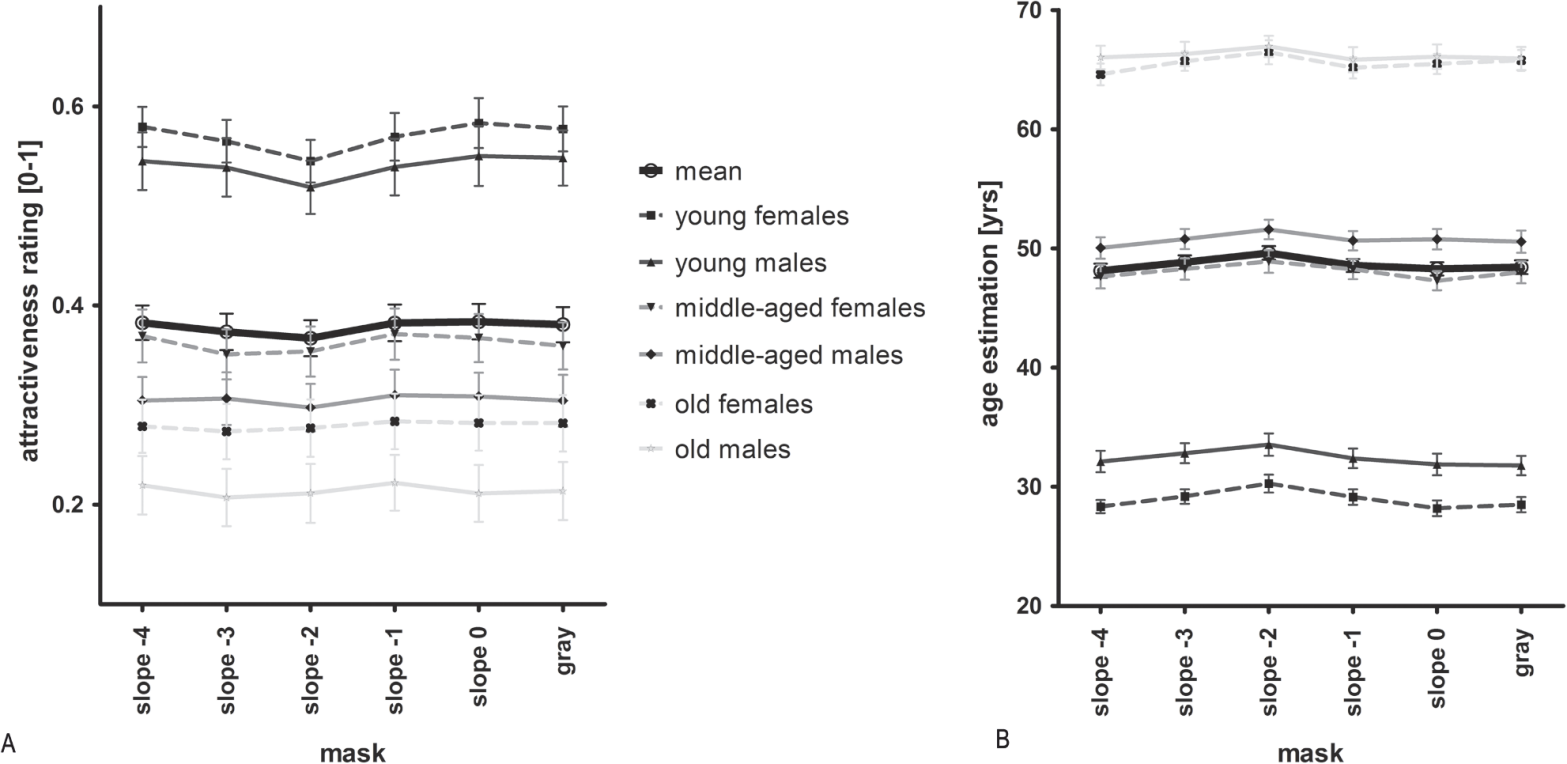
Results of attractiveness rating (A; N = 20) and age estimation (B; N = 21) in Study 2A. The different symbols and lines represent mean attractiveness or age ratings, respectively, for each stimulus category, as indicated, and the overall mean (thick solid line). Error bars represent standard error. For a statistical analysis, see text.

*Attractiveness rating*. We carried out a repeated-measures ANOVA with mask slope, stimulus age and stimulus gender as within-subject factors and found a significant influence of mask slope on attractiveness ratings (F(5, 95) = 8.775; p < .0001; Fig 3A). Faces with a mask with a slope of -2 were significantly less attractive than faces with a grey (control) mask or masks with a slope of -4, -1 or 0 (p < .05). Faces with a mask with a slope of -3 were also significantly less attractive than faces with a mask with a slope of -1 or 0 (p < .05). However, masks had different influences on the three age categories of the stimuli (young, middle-aged and old); the main effect was mainly driven by young faces. The different masks had only little effect on middle-aged and no effect on old faces (interaction: p < .01). On average, faces with a mask with a slope of -3 or -2 were rated less attractive (Fig 3). There were main effects of stimulus gender (F(1, 19) = 7.90; p < .05) and age category (Greenhouse-Geisser F(1.24, 23.49) = 67.11; p < .0001), i.e. female faces were rated as more attractive than male faces, younger persons more attractive than middle-aged and old persons, and middle-aged persons more attractive than older persons.

When we excluded the grey mask from the analysis and only analyzed the influence of the different slopes of the random phase patterns, we also found a significant influence of mask category that was fitted well by a quadratic relation (F(1, 19) = 16.853; p < .0001). Thus, faces with a mask with a median slope of -2 were rated least attractive compared to masks with more extreme slope values (Fig 3A).

*Age estimation*. Again, we ran a repeated measures ANOVA with mask, stimulus age and gender as within-subject factors and found a significant influence of mask slope on age estimations (Greenhouse-Geisser F(2.94, 58.76) = 14.76; p < .0001; Fig 3B). On average, faces with a mask with a slope of -2 were estimated as older compared to the other masks (p < .05). There were main effects of stimulus gender (F(1, 20) = 34.44; p < .0001) and age category (Greenhouse-Geisser F(1.44, 28.73) = 708.08; p < .0001), i.e. female faces were estimated as younger than male faces, younger persons as younger than middle-aged and old persons, and middle-aged as younger than old persons, as expected. On average, faces overlaid with a pattern with a slope of -2 were estimated 1.19 years older than in the control condition (grey mask). For young women, this effect was even larger (1.76 years older than control). Masks affected age estimation similarly in all stimulus categories (no significant interaction).

When we only analyzed the influence of the different slopes in the random phase patterns by excluding the grey mask from analysis, we also found a significant influence of mask category and a quadratic relation fitted best (F(1, 20) = 25.717; p < .0001). Thus, faces with a mask with a median slope of -2 were rated oldest compared to more extreme slopes (Fig 3B).

In sum, faces overlaid with a pattern with a slope of -2 and -3 were rated as less attractive and older, contrary to our hypothesis.

### Study 2B

#### Materials & Methods

*Participants*. Fifty participants (12 male, mean age = 22.1 years, range = 18–33 years) took part in Study 2B. All participants were students or graduates of medical or life sciences.

**Fig 4:**
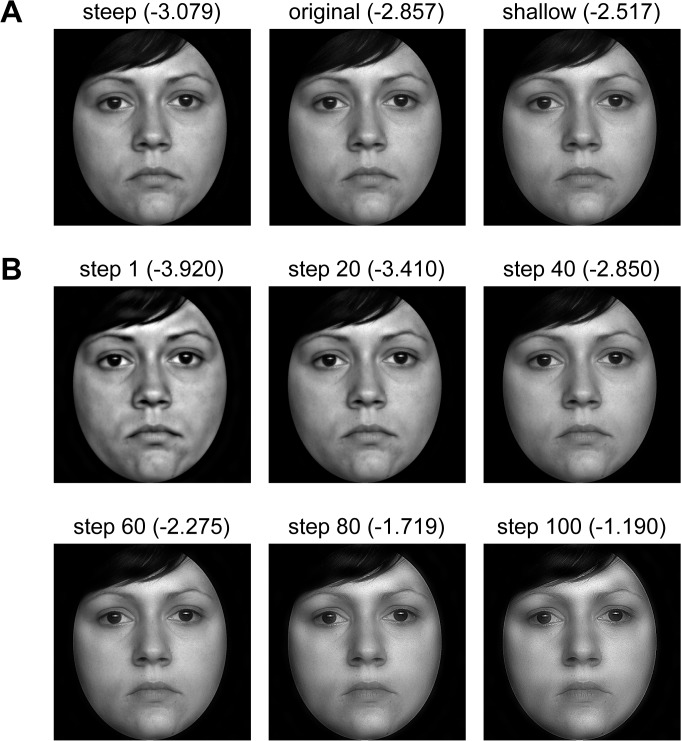
Example of the stimuli used in Studies 2B and 2C with corresponding Fourier slope in parentheses. Face images (from the FACES database [59]) with manipulated slopes of the radially averaged log-log Fourier power spectrum. A: Stimuli used in Study 2B. B: Stimuli used in Study 2C. Note that the subjective impression of each image changes with size and viewing distance, and that the images with shallower slopes become more blurry at this resolution due to the reduction of power in the low spatial frequencies. Images of original size are provided as supplemental material (S2 Fig and S3 Fig).

*Stimuli*. We used 40 images from the FACES database (see Study 1A). A black oval window was superimposed to hide external features, such as hair (see also Study 1A). Faces of persons of intermediate attractiveness between the age of 19 to 30 were used only (mean attractiveness rating = 2.56, SD = .27; results from Study 1A). The original images had a Fourier slope between -2.98 and -2.70 (M = -2.85; SD = .062; Table 5) and a resolution of 1024 x 1024 pixels. We increased or decreased the Fourier slope for every image with a Python-based algorithm. Due to technical limitations, it was not possible to obtain exactly the same slope value for each image. However, by measuring the slope of the manipulated images (see Study 1A), we confirmed that the manipulation made the slope either shallower (M = -2.585; SD = .032; paired-samples t-test: T(39) = -29.0, p < .0001) or steeper (M = -3.038; SD = .056; paired-samples t-test: T(39) = 16.7, p < .0001; Fig 4 and Table 5), respectively, than the original images. In total, we used 120 images (40 of each category: original, shallow and steep slope) as stimuli.

*Experimental design*. In every trial, two face images were shown simultaneously next to each other and participants were asked to decide which of the two faces was more attractive by pressing a corresponding arrow key on a keyboard. The two images shown in every trial had different slopes (shallow, original, steep) and were from *different persons*. Images were shown at a viewing distance of 100cm that was ensured by the use of a chin rest. Each image was presented at a maximum size of 6.1° vertical visual angle. A question mark was presented first (800ms), followed by the face images (600ms), and a blank screen (1600ms). As soon as the images were shown, the participants were free to respond within 2200ms. After 80 trials, the participants were allowed to take a break of any length before evaluating the next images. Every participant viewed 600 trials in total.

#### Results

Regardless of image pairing within a trial, original face images were chosen in 34.19% of trials (SD = 1.74), faces with a shallow slope in 34.22% of trials (SD = 3.22) and faces with a steep slope in 31.49% (SD = 3.14) of trials (Table 6). We compared the absolute rates of each individual face image in each category (original, steep and shallow slopes; Table 6) with a repeated measures ANOVA. We found a significant influence of category on attractiveness ratings (F(2, 78) = 15.109, p < .0001). Face images with a steep slope were chosen significantly less frequent than faces with a shallow slope or the original faces (both comparisons p < .001). We found no difference in ratings between faces with a shallow slope and the original images.

**Table 6:** Summary of results of Study 2B.

image paired with →	**steep slope**	**original**	**shallow slope**	**paired-samples t-test**		**total** *	**absolute rating**
↓ image chosen	(-3.04)	(-2.85)	(-2.59)	T	p		
**steep slope (-3)**	x	47.87%	46.58%	1.847	.071	31.49%	235.53
**original (-2.8)**	52.13%	x	50.41%	-1.484	.144	34.19%	255.68
**shallow slope (-2.5)**	53.42%	49.59%	x	-4.968	< .0001	34.32%	256.73

* mean frequencies of selecting that image, regardless of pairing. For further information see text.

Images with a shallow slope were selected as more attractive more often when paired with an image with a steep slope then when paired with the original images (paired-samples t-test: T(49) = -4.968, p < .0001; Table 6). There was a tendency that participants chose the face image with the steep slope more often when paired with the original image than when paired with a shallow slope image (T(49) = 1.847, p = .071). We found no difference in selection frequency of the original face images when paired with images with a steep slope, or when paired with images with a shallow slope (T(49) = -1.484, n.s.).

In summary, these results reflect a preference for original face images and those with a shallow slope compared to those with a steep slope.

### Study 2C

#### Materials & Methods

*Participants*. Twenty-one participants (10 male, mean age = 23.3 years, range = 19–30 years) took part in Study 2C. All participants were students or graduates of medical or life sciences.

*Stimuli and Procedure*. We used 120 images (60 male, 60 female) from the FACES database with a black oval window (see Study 1A). The images had a resolution of 512x512 pixels and were presented at a size of 6.1° of vertical visual angle. With the aid of a PsychoPy program (v1.78.01) [71], participants changed interactively the Fourier slope. The slope was adjusted by moving a computer mouse on a free scale. For each face, the stimuli consisted of 100 images with slope values that ranged from about -4 to -1 in about equally sized steps (Fig 4 and Table 5). The slope between two consecutive images changed by an average of. 027 (SD = .005). Participants were asked to adjust the image (by changing its slope) until the face looked as attractive as possible. The slope was set to a random value before each trial and the actual slope value and the mouse cursor were not visible to the participants during the adjustment.

#### Results

When asked to select the most attractive face, participants adjusted the images to mean slope values of -2.643 (SD = .224). The adjusted slope was significantly shallower than the slope of the original images (M = 2.755, SD = .116; paired-samples t-test T(119) = -7.148, p < .0001; Table 5). For 89 of the 120 stimuli, participants adjusted the slope so that it was shallower than the original slope.

Interestingly, the effect differed according to gender and age. For gender, the original slope was similar (females: M = -2.756, SD = .116; males: M = -2.753, SD = .117; two-samples t-test: T(118) = .058, p = .881). The adjusted slope did not differ significantly between genders although female faces were adjusted to a shallower slope (females: M = -2.611, SD = .235; males: M = -2.677, SD = .207; two-samples t-test: T(118) = -1.623, p = .206). However, the size of the reported effect was significantly stronger for female faces (two-samples t-test: T(118) = 2.162, p < .05). For age, face images differed in original slope (young: M = -2.821, SD = .081; middle-aged: M = -2.674, SD = .101; two-samples t-test: T(118) = -8.790, p < .0001) and adjusted slope (young: M = -2.770, SD = .160; middle-aged: M = -2.486, SD = .189; two-samples t-test: T(118) = -8.872, p < .0001). The reported effect was significantly stronger for old faces (two-samples t-test: T(118) = 4.722, p < .0001).

### Discussion

In the experiments of Studies 2A, 2B and 2C, we manipulated the Fourier power spectrum characteristics of face images.

In Study 2A, mean chronological age of male and female stimuli did not differ (Table 4; Mann-Whitney-U-test: Z = -.079; n.s.) but age estimation did, with female faces being estimated younger than male faces. This finding corresponds to the results by Voelkle and colleagues [72] who observed that male faces are estimated older than their true age while female faces are estimated younger.

Participants estimated face images with a mask with a slope of -2 on average 1.2 years older than in the control condition (i.e., grey mask). A change of similar size was reported in a previous study on the influence of facial contrast on age perception [20].

Face images overlaid with a random phase pattern with a Fourier slope of -2 (and -3) were rated as less attractive and perceived as being older. However, we expected higher attractiveness ratings with either a mask with a slope of -2 or with stimuli that have a shallower slope overall (i.e., overlaid slopes as -1 or 0). A possible explanation for this discrepancy might be that we introduced additional structure to the face images by overlaying random phase patterns. This additional information might have caused faces with masks with a slope of -3 and -2 to subjectively look more unhealthy (see Fig 1) and this impression might affect both age and attractiveness perception [11, 13, 68]. Interestingly, patterns with slopes that are not common in nature (i.e., slopes 4, -1 and 0) had no influence on age and attractiveness ratings in this study (compared to the grey control mask). However, changes in the slope of the resulting images between stimulus categories were quite small (Table 5).

The mask affected the attractiveness rating mainly of young faces but less so of older faces. A possible explanation for this finding is that a mask with a slope of -2 leads to a dappled face surface that is more visible in younger faces because younger faces have a smoother skin texture than in older faces in general. However, a mask with a slope of -2 induced a similar change in age perception across all stimulus age categories. While a dappled surface might affect age evaluation in all age categories, attractiveness of older faces does not seem to be affected by this manipulation, possibly because perceived attractiveness of older faces was low already. As an alternative explanation, we note that participants were young (between 18 and 30 years old) and attractiveness perception might be affected most strongly in faces within the raters' own age group because they constitute potential mates or rivals.

Because the superposition of random structure onto face images in Study 2A might have had an effect on skin perception, we directly manipulated the slope of the face image without introducing additional information in the Studies 2B and C. Participants either assessed the same faces with modified Fourier slopes (but never the same face in direct pairwise comparison; Study 2B) or they manipulated the Fourier slopes of single face images interactively (Study 2C). Therefore, we measured pure effects of image manipulation.

In Study 2B, subjects chose images with a steep slope less often, indicating a disfavour of a steep slope in the face images. The small size of these differences can be explained by the fact that changes within the image were very subtle. Moreover, participants did not decide between two variations of the same face but between the faces of two persons who differed in attractiveness in most cases.

In Study 2C, face images with a shallower slope than the original images were perceived as most attractive. Blurring or smoothing of the skin, which photographers use do to render portraits more aesthetic, cannot explain our results because fine detail is enhanced in images with a shallower slope. Our finding is compatible with the hypothesis that images with statistics more similar to natural scenes are perceived more efficiently and/or fluently (see Introduction) and, consequently, as more beautiful (or more attractive in the case of faces). This notion is in line with findings of facilitated face recognition (reaction time and ERP measures) in images with shallow slopes compared to steep slopes [50]. Interestingly, in our study the effect was significantly larger for female faces, meaning that participants adjusted female face photographs to a shallower slope than male ones, although original images did not differ. One explanation might be that in images with a shallower slope, eye brows are less prominent (due to decrease of LSF power) and, thus, female faces become more attractive [73].

In Studies 2B and C, we did not add any new phase or other information to the images, but only changed the relative prominence of the spatial frequencies. Nevertheless, the subjectively perceived skin texture of the faces apparently changes with slopes values that are exceedingly steeper than -3. We offer two possible explanations for our findings. First, with steeper slopes, the faces become dappled (Fig 4), which might be a reason why participants do not find these faces attractive, perhaps due to an unhealthy appearance of the skin. Second, previous studies demonstrated an increase of discomfort after lowering the amplitude of HSFs in random phase patterns (e.g., [74]).

## Study 3

In this study, we did not modify the face images themselves but changed the statistical information provided by the background of the face images. Specifically, we investigated whether and how different statistics of a random-phase background can influence attractiveness of the face in the foreground. By changing the background only, we excluded any—however small—change in the visual appearance of the face. We expected that a background with a pattern similar to the structure of natural scenes and artworks (slope of -2) would have a positive effect on facial attractiveness.

### Study 3A

#### Materials & Methods

*Participants*. Originally, 30 participants took part in this study. Data of two of the participants were removed from the analysis because they evaluated only about half of the stimuli. Hence, data from 28 participants (five males, mean age = 23 years, age range 18–28 years) were used for analysis. Participants were mainly students and graduates from different fields.

**Fig 5:**
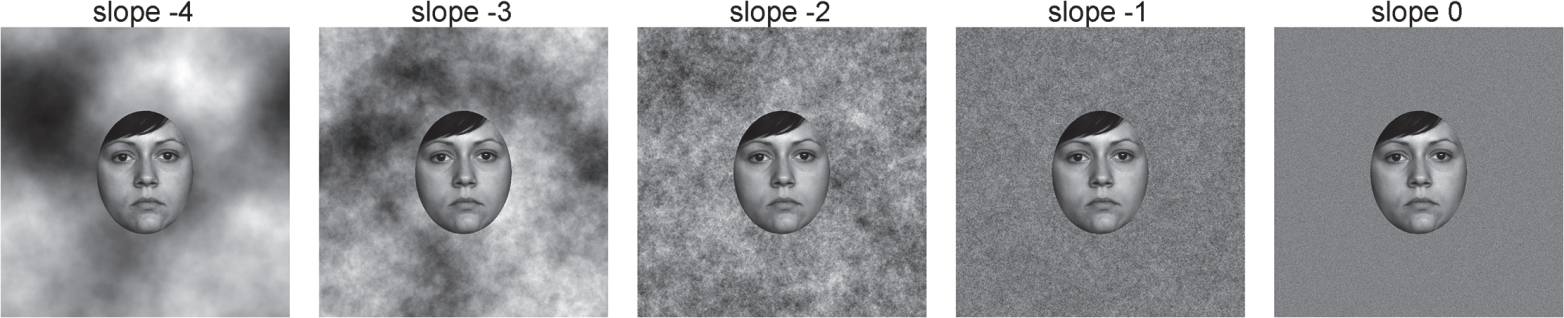
Stimuli used in Study 3A. Faces from FACES database [59] in front of random phase patterns with different slopes in the radially averaged log-log Fourier power spectrum.

*Stimuli*. We used 100 photographs from the FACES database (see Study 1A). The photographs were images of 50 females and 50 males. For each gender 25 persons were young (19–30 years) and 25 persons were middle-aged (39–55 years), respectively. Oval cut-outs of the face images were presented in front of random phase backgrounds of five different slope values (-4, -3, -2, -1 and 0; Fig 5). We generated 100 different background images per slope (see Study 2). For each slope value, each face was presented in front of a randomly selected background image, i.e. each face was shown five times. Participants viewed stimuli at 17.1° x 17.1° of the visual angle and faces covered 5.7° x 8.4°.

A representative number of stimuli (each face in front of ten different random phase patterns per slope category; i.e., 5000 images) were analyzed using Fourier transformation (see Study 1A). As expected, the background had a strong effect on the Fourier slope of the entire image (Greenhouse-Geisser F(1.56, 154.27) = 54850.8, p < .0001, all post-hoc pairwise comparisons: p < .0001; Table 5).

*Experimental design*. Participants were asked to rate the attractiveness of a presented face on a 4-point-scale (1 not attractive, to 4 very attractive) per keystroke. Since each face (N = 100) was presented in each condition, participants run 500 trials. Stimuli were presented in random order. A question mark was presented first (1000ms), followed by the stimulus (800ms), and a blank screen (1700ms). As soon as the images were shown, the participants were free to respond within 2500ms. Participants were allowed to take a break of desired length after 62 or 63 trials before evaluating the next block of images.

#### Results

**Fig 6:**
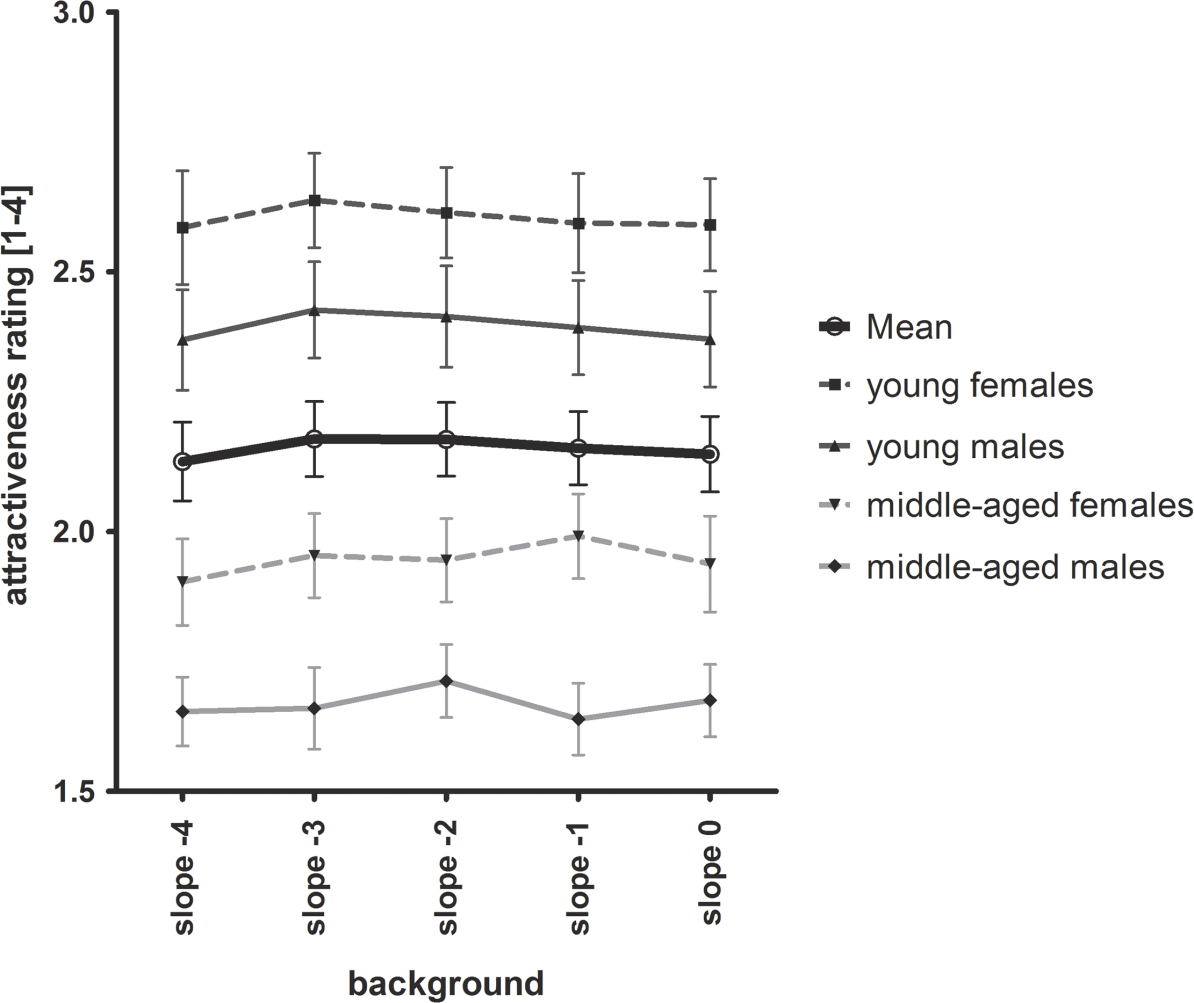
Results of Study 3A. Different symbols and lines represent different stimulus categories and the overall mean (thick solid line). Error bars represent standard-error. For a statistical analysis, see text.

The different Fourier statistics of the backgrounds significantly influenced attractiveness ratings (repeated-measures ANOVA: F(4, 108) = 3.12; p < .05; Fig 6). This relationship was quadratic (F(4, 27) = 8.76; p < .01). Faces in front of patterns with median slopes (i.e., -2) received higher attractiveness ratings than faces in front of extreme slopes. Additionally, there was a significant difference of attractiveness ratings between the faces in front of backgrounds with a slope of -4 and -2 (post-hoc test: p < .05). Furthermore, we found main effects of stimulus age (F(1, 27) = 137.43; p < .0001) and gender (F(1, 27) = 18.05; p < .0001; Fig 6) but no interactions with the slope of the background image; none of the interactions was significant. Thus, as in Study 2, female faces were rated as more attractive than male faces, and younger faces were rated as more attractive than faces of older persons.

In addition to our main analysis, we analyzed averaged rating data of each participant by calculating the rank of mean attractiveness ratings for the different backgrounds for each participant. Again, results show a significant effect of background category on attractiveness ratings (Friedman test: *X* ^*2*^(4) = 17.537, p < .01). Pairwise comparisons with Sign tests revealed that faces in front of patterns with a slope of -3 and -2 were rated as significantly more attractive than faces in front of patterns with shallower slopes (-1 and 0; Z_max_ = -2.08; p < .05). Also, there was a tendency that faces in front of backgrounds with slopes of -3 and -2 were rated as more attractive than in front of patterns with a slope of -4 (Z = -1.93, p = .054 and Z = -1.77, p = .078, respectively).

### Study 3B

#### Materials & Methods

It is possible that the beauty of backgrounds is perceived differently and, thus, faces were evaluated accordingly. To further study whether the background had a direct (e.g. contrasting or reinforcing) effect on the face attractiveness ratings in Study 3A, we asked the 41 subjects, who had participated in Study 2A before, to evaluate the beauty of the random phase patterns used in Study 3A (without a face in front of it; examples are shown in Fig 1, top row) and the mid-grey image of Study 2A. Stimuli were presented at a size of 17.1° x 17.1° of visual angle. Each image was shown once in a total of six trials. Participants rated the beauty of these images on a mouse-based continuous-looking rating scale with 100 sub-steps (not beautiful *versus* beautiful). The mouse cursor was set to the midpoint of the scale before each rating. After a fixation cross was presented for a random length between 300 to 800ms, test images appeared for 800ms. Then participants had time to respond without a time limit.

#### Results

Participants perceived the beauty of the random phase patterns differently (Friedman test: *X* ^*2*^(5) = 44.73, p < .0001). Beauty ratings were highest for images with slopes of -4 and -3, and then dropped with shallower slopes until lowest values were reached for images with a slope of 0 and the mid-grey images, respectively (Table 7).

**Table 7:** Summary of results of Study 3B and pairwise comparisons (Wilcoxon tests).

	grey	slope 0	slope -1	slope -2	slope -3	slope -4
mean	.2383	.2190	.2922	.3812	.4439	.4320
SE	.0395	.0314	.0373	.0342	.0333	.0394
grey		Z = -.094, p = .929	Z = -1.734, p = .083	Z = -2.897, p < .01	Z = -3.623, p < .001	Z = -3.335, p < .001
slope 0			Z = -2.351, p < .05	Z = -3.584, p < .001	Z = -4.303, p < .0001	Z = -3.771, p < .0001
slope -1				Z = -2.190, p < .05	Z = -3.657, p < .001	Z = -3.072, p = .01
slope -2					Z = -2.002, p < .05	Z = -1.701, p = .090
slope -3						Z = -.624, p = .539

### Discussion

In this study, we did not modify the face images themselves but their background. Nevertheless, we found differences in attractiveness ratings (Study 3A). Faces in front of backgrounds with statistics more similar to natural images (slope of -2, natural scenes; and slope of -3, faces) were perceived as more attractive compared to face images with the other backgrounds. This result is compatible with the hypothesis that images with statistical properties similar to those of natural images are perceived more efficiently and/or fluently and are, thus, perceived as more beautiful or attractive (see above).

In a previous study on visual discomfort by Juricevic et al., subjects disliked random phase patterns with a steep slope (-4) most; lowest discomfort was found for images with a slope of -2 [31]. The response pattern of the study by Juricevic et al. could, theoretically and in part, explain the results of Study 3A through a reinforcing effect [31]. However, we can exclude contrasting or reinforcing effects in Study 3A because our beauty ratings of the background images revealed a different pattern of preference than the foreground face images (Study 3B). The differences between the results of the study by Juricevic et al. and our Study 3B may also be due to differences in the experimental set-up (presentation time, size of and distance to stimuli, beauty *versus* discomfort rating) [31]. Random phase patterns with a steep slope (i.e., -4 and -3) might induce discomfort because of perceived blur. However, in these images compared to those with shallower slopes, some naturally looking structure can be perceived and might have led to higher beauty ratings. The finding that beauty ratings were highest for patterns with slopes of -4 and -3, however, does not support the aforementioned hypothesis that images with statistics similar to natural scenes are perceived as more beautiful because they are processed more efficiently and/or fluently. A random phase pattern with a slope of -2 might not be beautiful *per se* because not all such images are beautiful [37]. However, in combination with a face image, a slope of -2 might influence neural processing of the face and, consequently, the faces may be rated as more attractive.

As an alternative explanation, we suggest that the aesthetic (or more attractive) evaluation is due to a match of the statistics of fore- and background because the face images, which possessed a slope of about -2.80 (SD = .10), were rated as more attractive in front of backgrounds with a similar slope (-3 and -2).

We can only speculate about the neural mechanisms behind the effect of different background statistics on perceived face attractiveness. If two stimuli (face and background in our case) are presented simultaneously within the visual field, these stimuli are not processed independently (as reviewed in [75]). The resulting sensory competition, however, might be biased in our study because participants attended to one of the stimuli only (i.e., the face; top-down biased competition) (as reviewed in [75]). Competition between face and background might be highest with background slopes of -3 and -2 because of the statistical similarity to the face images. Further investigations are needed to examine the neural mechanisms underlying our results.

## General Discussion

In the present work, we investigated the relation of facial attractiveness and age perception to statistical image properties. To this aim, we studied properties that are shared by images of natural scenes and artworks (including art portraits). In unaltered face images, Fourier slope did not predict attractiveness ratings, and PHOG self-similarity correlated negatively with attractiveness ratings when measures were controlled for the effect of age (Study 1). These findings contradict our hypothesis that natural scene statistics in the Fourier domain (i.e., a slope of -2) and self-similarity correlate positively with the attractiveness of the depicted faces. However, in the experimental part of the present study face images with shallower slopes (i.e., a shift towards slopes of natural scenes) were liked most if the Fourier slope was modified directly in the face image (Study 2B). When a random phase background surrounded the faces (Study 3A) faces were rated as more attractive in front of random phase images with statistics similar to natural scenes (i.e., slope of about -2) or similar to face images (i.e., slope of about -3). In Study 2C, participants directly manipulated the Fourier slope and adjusted it to make the face images look as attractive as possible. Results revealed that the adjusted face images had a shallower slope than the original images (reflecting a relative increase of HSF power). The effect was significant for both genders but was stronger for face photographs of females. As expected when evaluating images of the same faces—compared to evaluating images of different groups of faces—the observed effects were small, but there were significant.

Results from Studies 2B, 2C and 3A support both the efficient coding and fluency hypothesis. We assume that images with natural (or 'matching') statistics are processed more efficiently and/or fluently and, thus, were judged as more beautiful (see [41, 53]). In recognition and categorization studies, natural scenes statistics led to better performances [50, 51]. Blickhan et al. [50] found that neural correlates of face representation were facilitated when faces were learned with face images with a slope of -2. This type of shallow slope (i.e., increased HSF power) not only reinforces face learning but may also have a positive effect on facial attractiveness. From a different point of view, O'Hare and Hibbard suggested several—not mutually exclusive—explanations for visual discomfort [32]. One of their explanations, i.e. deviation from natural scenes statistics [30, 31], is compatible with the results from our Studies 2B, 2C and 3A. Additionally, hyperexcitation of the visual system and accommodation failure due to blurred images [74, 76] are other possible explanations that cannot be confirmed or ruled out by our data.

The present study was a first investigation to relate Fourier power spectrum characteristics of face images to facial attractiveness and age perception. We demonstrate that attractiveness ratings, although highly dependent on morphological characteristics (e.g., [8]), are also affected by statistical image properties that are not face-specific and have been studied in other types of images previously, for instance by using spatial frequency modifications. Interestingly, a change in attractiveness evaluation was observed even if the image of the face itself was not modified (Study 3A) and, consequently, other indicators of attractiveness, such as symmetry, averageness, secondary sexual characteristics, and skin texture remained constant. In conclusion, our results provide evidence that perceived facial attractiveness (and age) correlate with and can be modulated by low-level image properties that are supposedly processed at early stages of visual perception. However, the exact neuronal mechanisms underlying our results remain to be investigated.

## Supporting Information

S1 Fig Images of Fig 1 in the manuscript in original resolution. Top row: Random phase patterns with different slopes of the radially averaged Fourier power spectrum, and a mid-grey control image. Bottom row: Stimuli used in Study 2A. The composite stimuli consists of the original face image (FACES database [59]) with the respective image of the top row superimposed at an opacity of 15% and a black oval window.
(TIF)

S2 Fig Images of Fig 4A in the manuscript in original resolution. Example of the stimuli used in Study 2B. Face images (from the FACES database [59]) with manipulated slopes of the radially averaged log-log Fourier power spectrum.
(TIF)

S3 Fig Images of Fig 4B in the manuscript in original resolution. Example of the stimuli used in Study 2C. Face images (from the FACES database [59]) with manipulated slopes of the radially averaged log-log Fourier power spectrum.
(TIF)
