# Supplementary figures and images for: Fourier Power Spectrum Characteristics of Face Photographs: Attractiveness Perception Depends on Low-Level Image Properties
Source: PLoS One. 2015 Apr 2;10(4):e0122801. doi: 10.1371/journal.pone.0122801 (PMC4383417; doi:10.1371/journal.pone.0122801)

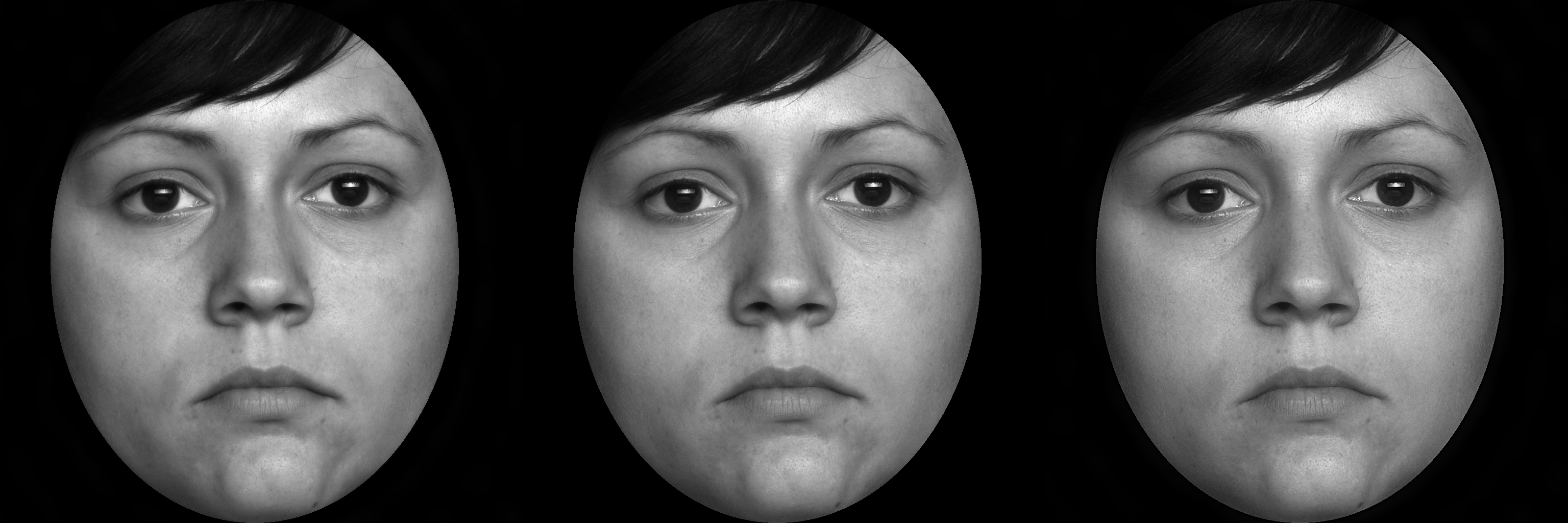

Supplement: S2 Fig — Example of the stimuli used in Study 2B. Face images (from the FACES database [59]) with manipulated slopes of the radially averaged log-log Fourier power spectrum. (TIF) [file pone.0122801.s002.tif]
